# Diagnostic Workup and Treatment of a Rare Apocrine Hidrocystoma Affecting the Oral Mucosa: A Clinical and Histological Case Report

**DOI:** 10.1155/2017/9382812

**Published:** 2017-07-11

**Authors:** Pier Paolo Poli, Luca Creminelli, Valeria Moramarco, Alessandro Del Gobbo, Franco Ferrante, Carlo Maiorana

**Affiliations:** ^1^Oral Surgery Unit, Department of Maxillofacial Surgery and Odontostomatology, Fondazione IRCCS Cà Granda Ospedale Maggiore Policlinico, Via Commenda 10, 20122 Milan, Italy; ^2^Department of Anatomic Pathology, Fondazione IRCCS Cà Granda Ospedale Maggiore Policlinico, Via Francesco Sforza 35, 20122 Milan, Italy; ^3^Oral Surgery and Implantology Units, Department of Maxillofacial Surgery and Odontostomatology, Fondazione IRCCS Cà Granda Ospedale Maggiore Policlinico, University of Milan, Via Commenda 10, 20122 Milan, Italy

## Abstract

Apocrine hidrocystomas are rare benign cystic tumors originating from the secretory portion of apocrine sweat glands. To the best of our knowledge, there is no evidence currently available reporting the presence of apocrine hidrocystomas in the oral cavity. Therefore, this case report aims to describe the clinical and histological features of an apocrine hidrocystoma affecting the oral mucosa. A 69-year-old male patient presented with a 1-year history of a solitary, well-circumscribed, submucosal mass in the left posterior buccal mucosa. The clinical examination revealed a yellowish soft, fluctuant, and painless lesion with no clinical signs of erythema or ulcerations of the overlying epithelium. The entire lesion was excised and histopathological analysis confirmed the diagnosis of apocrine hidrocystoma. No recurrence was observed after a 1-year follow-up.

## 1. Introduction

Hidrocystomas are rare, benign, cystic tumors of sweat glands conventionally classified into apocrine and eccrine types according to their histological characteristics and presumed histogenetic derivation. Apocrine hidrocystoma (AH) has been described as an adenomatous cystic proliferation of the secretory coil of apocrine glands [1]. Clinically, AH appears as a solitary, well-defined, dome-shaped nodule with a smooth surface and variable colours, ranging from flesh colour to blue-black colour [2]. Although the most common presentation is a solitary lesion, instances of multiple AHs have been reported [3]. The typical localization of AHs is the periorbital area; however they may also occur on the lips, ears, neck, scalp, chest, shoulders, or feet. Very rarely AHs are documented to be on axilla, penis, and anus as well [4]. Histologically, AH is characterized by the presence of one to several layers of cuboidal or columnar cells showing decapitation secretion and expression of a keratin pattern of secretory type [5]. Treatment options for solitary hidrocystomas may vary from simple needle puncture or surgical excision to electrodessication, anticholinergic creams, carbon dioxide vaporization, and laser treatment depending on the solitary or multiple nature of the lesion, respectively [2].

To the best of the authors' knowledge, this could be the first case in literature reporting the clinical and histological features of an AH affecting the oral mucosa. However, this is difficult to ascertain due to the complex terminology in this field reporting abundant and repetitive terms used over time to indicate similar lesions.

## 2. Case Presentation

A 69-year-old male patient reported with a chief complaint of chewing difficulties due to a soft tissue mass in the left buccal mucosa, which had been present for 1 year. The cervical lymph nodes were not palpable and there was no swelling in the overlying skin. The medical records were noncontributory except for primary hypertension controlled with antihypertensive drugs.

**Figure 1:**
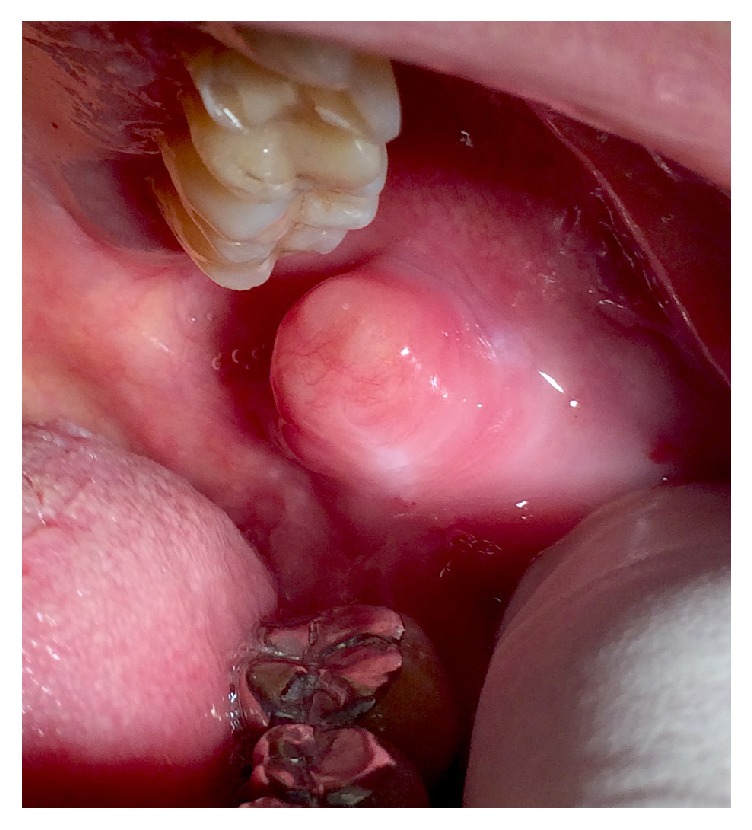
Intraoral aspect of the lesion located in the left posterior buccal mucosa near the retromolar triangle. The clinical examination revealed a yellowish well-circumscribed, movable submucosal mass of approximately 21 × 15 mm in diameter, covered by a normal mucosa.

The clinical intraoral examination revealed a solitary, well-defined, movable, exophytic submucosal mass 2 mm below the opening of Stensen's duct. On palpation, the lesion was soft, fluctuant, and painless with clear margins. The mass had a yellowish appearance and the overlying mucosa was normal with no clinical signs of erythema or ulcerations. The overall clinical appearance of the mass suggested a benign lesion (Figure 1).

Because of the need of a histopathologic analysis to confirm the final diagnosis, the surgical removal of the lesion was chosen among the possible therapeutic options. After bacterial decontamination with an antiseptic rinsing solution of 0.2% chlorhexidine digluconate for 1 minute, local anaesthesia was obtained by infiltrations of mepivacaine 2% with epinephrine 1 : 100.000. The cheek was then everted with digital pressure to increase the lesion's prominence. The lesion was clamped with Klemmer forceps and an elliptical half-moon incision was performed at the base. Flap was reflected cautiously with blunt dissection to avoid any tissue rupture. Thus, the buccal mucosa was undermined exposing an irregular, poorly encapsulated, and lobulated yellowish mass. The lesion was periosteum-free with no attachment to any contiguous structures including the mandibular bone. The entire lesion was then carefully dissected from the surrounding soft tissues and excised en bloc. A tension-free primary intention closure was accomplished with resorbable 4-0 single stitches. Stensen's duct papilla was identified and saliva was expressed by digital manoeuvre, confirming that it was not damaged. The excised specimen was sent for histopathological examination.

**Figure 2:**
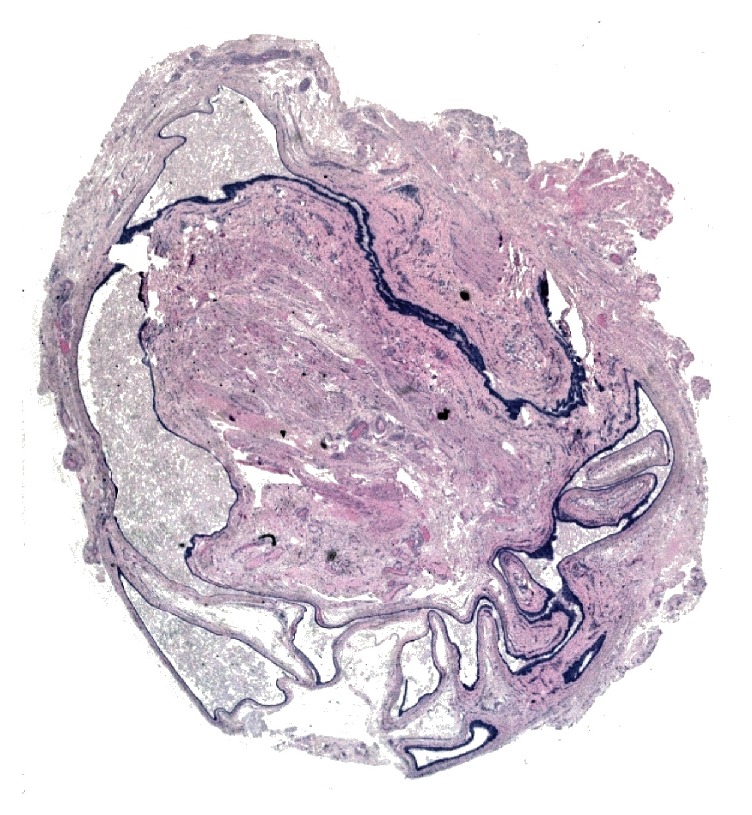
Low-power magnification of the resected specimen measuring roughly 19 × 13 × 8 mm, showing an encapsulated solid lesion characterized by a multilocular cystic space with fibrous tissue lined by a stratified epithelium. In the cystic lumen, proteinaceous fluid is present (haematoxylin and eosin; original magnification ×10).

**Figure 3:**
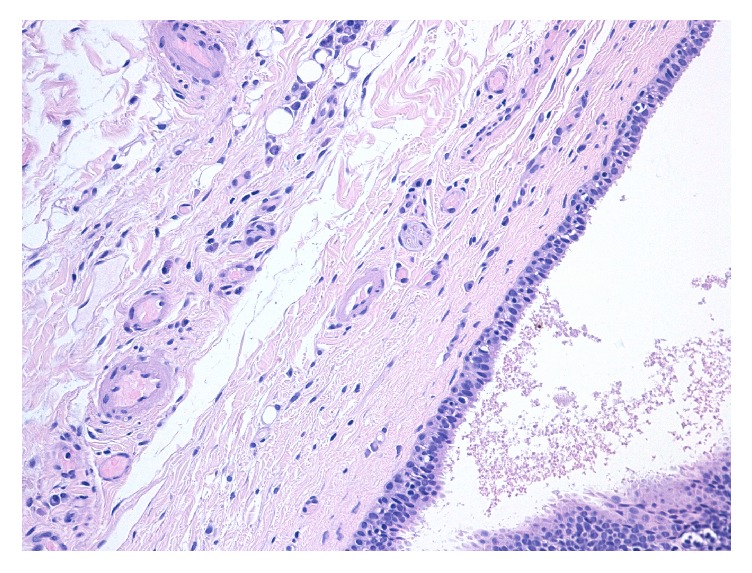
More details of the cystic wall composed of fibrous tissue with its surrounding stroma containing microvessels and apocrine-like secretory epithelium consisting of epithelial sweat-duct-like lining without cytologic atypia (haematoxylin and eosin; original magnification ×200).

**Figure 4:**
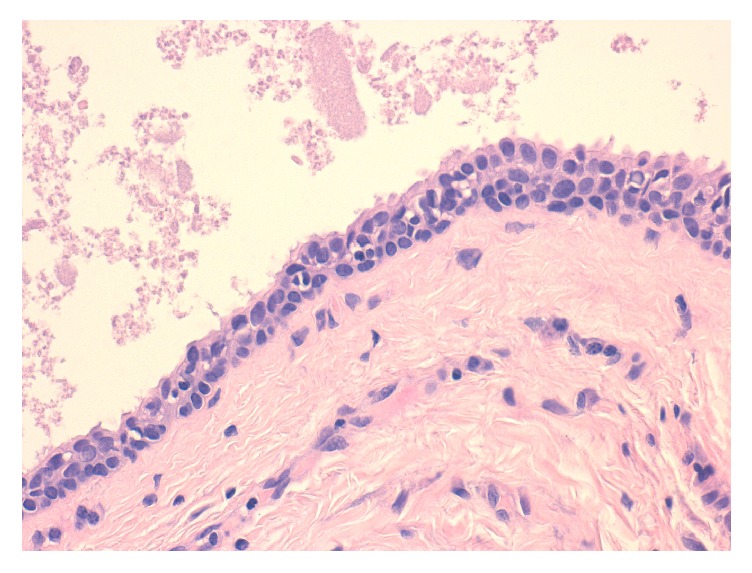
Higher magnifications of the cystic wall composed of a stratified apocrine-like secretory epithelium consisting of epithelial sweat-duct-like lining without cytologic atypia. Rows of secretory cells showing decapitation secretion characterized by apocrine snouts and fringes are clearly recognizable (haematoxylin and eosin; original magnification ×400).

**Figure 5:**
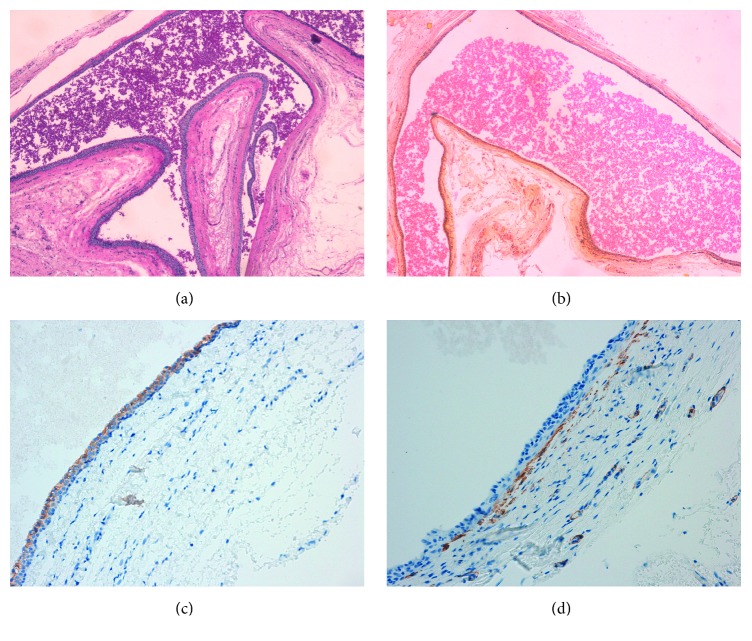
Histopathological staining. (a) Periodic Acid Schiff histochemical staining, showing PAS-positive content of the cyst (original magnification ×50); (b) mucicarmine staining highlighting the mucinous content of the lesion (original magnification ×50); (c) human epidermal growth factor receptor 2 immunohistochemical staining, showing diffuse cytoplasmic expression and confirming the secretory nature of the epithelial lining (original magnification ×200); (d) smooth muscle actin immunohistochemical staining highlighting myoepithelial cells (original magnification ×200).

From an initial macroscopic evaluation, the resected specimen consisted of a well-circumscribed and yellowish mass with a soft consistency. The histopathologic examination showed a multiloculated cyst characterized by a stroma composed of fibrous tissue with dilated blood microvessels. In the cystic lumen, proteinaceous fluid was present (Figure 2). Microscopically, the lesion consisted of a cystic wall lined by two or more rows of sweat-duct-like epithelium, with monomorphous cubic cells characterized by a small, central nucleus and without cytologic atypia (Figure 3). The cubic cells showed decapitation secretion indicative of apocrine secretion (Figure 4). Normal glands as well as adnexal structures or connections to dermis of the overlying skin were not identified in the histological sections. The lesion was then subjected to immunohistochemical staining to support the diagnosis (Figures 5(a)–5(d)). All antibodies were provided by DAKO and antigen-antibody detection was performed with the DAKO Omnis automated staining platform (DAKO A/S, Glostrup, Denmark) according to manufacturer's instructions. To study the mucin expression, the intraluminal secretion was analysed with the Periodic Acid Schiff (PAS) reaction with and without diastase and with mucicarmine staining, both of which were positive. Further, to demonstrate the apocrine secretory nature of the luminal cells, human epidermal growth factor receptor 2 (HER2) expression was observed. In addition, immunohistochemistry showed that the basal cells of the cystic wall were positive for smooth muscle actin (SMA) confirming their myoepithelial origin. A final diagnosis of apocrine hidrocystoma was established. The resection margin was negative. No recurrence was observed over a 12-month follow-up.

## 3. Discussion

Apocrine hidrocystomas are benign cystic tumors of the apocrine sweat glands. The estimated incidence is one per thousand of submitted cutaneous biopsies; however the true value remains unknown and underestimated since many times apocrine hidrocystomas are seen in ophthalmology or surgery clinics or are diagnosed as Moll's gland cysts when located on the inner or outer canthus of the lower eyelid [6]. Although different locations of presentation have been reported, to our knowledge there is no evidence of apocrine hidrocystomas affecting the oral cavity.

With respect to the etiopathogenesis, the lesion could be correlated with a trauma to the oral mucosa of the cheek during a masticatory act. This supports the hypothesis that AHs may be a consequence of an occlusion or blockage of the sweat-duct apparatus, resulting in retention of sweat and a dilated cystic structure. Apparently, the clinical features of the oral AH resembled those reported for the same lesion in different anatomical districts. It appeared as a slow-growing dome-shaped, solitary, well-delimited nodule with a cystic consistency. Though the most common presentation is a solitary lesion, some cases of multiple apocrine hidrocystomas have been described often in association with Gorlin-Goltz and Schopf-Schulz-Passarge syndromes [4]. The lesion may assume various colours including light brown, red-brown, bluish, black, or skin colours; however in the oral cavity a yellowish colour was observed. According to the present report, most cases reported a diameter < 20 mm ranging mostly from 3 to 15 mm, [2] although “giant” AHs of ≥20 mm have been rarely documented [7, 8]. The age presentation in the present report corroborates the range reported in literature. Indeed, apocrine hidrocystomas are prevalent in adults between 30 and 75 years of age with equal sex incidence [9]. Considering these features, the clinical differential diagnosis included lipoma and fibrolipoma which are both frequently well-circumscribed and encapsulated and often exhibit capillary vessels in the overlying mucosa. Other similar intraoral lesions comprise mucoceles, fibromas, fibrous hyperplasia, lymphangioma, and salivary gland lesions; however the final diagnosis is provided by histopathologic analysis.

Microscopic examination showed that the lesion contained multilocular spaces while a sweat-duct-like epithelium with monomorphous cubic cells was observed. Further, the cyst wall was covered by apocrine-type epithelial cells with marked decapitation secretion identified by apocrine snouts. No cytologic atypia was detected. This histological pattern of the lesion has been similarly described in its skin counterpart. Indeed, the AH has been reported to be encapsulated, well-circumscribed with surrounding fibrotic tissue, and often multiloculated. Microscopically, cystic ducts are lined mainly by a double-layered epithelium, with no evidence of squamous or mucous metaplasia [9]. A pathognomonic sign is the presence of large columnar or cuboidal cells in the composition of the epithelial lining showing a decapitation secretion, usually associated with an outer layer of flat myoepithelial cells [9, 10]. Immunohistochemistry was used to strengthen the diagnosis. We performed immunohistochemistry using DAKO Omnis platform in order to guarantee a high degree of standardization of the staining. Total automation of this system allowed obtaining all the staining needed at the same time, avoiding preanalytical biases. In particular, PAS and SMA positivity has been associated with apocrine differentiation [2, 4, 5, 9].

Although the lesion was asymptomatic the patient complained of impaired chewing abilities, which have a negative impact on the oral health related quality of life. For this reason, the mass was completely excised to reestablish a physiologic occlusion immediately after the surgical procedure and to prevent recurrence chances. This was accomplished through a split-thickness incision and blunt dissection followed by a tension-free suture. Other methods have been reported to treat intraoral lesions including marsupialization, CO_2_, and Er,Cr:YSGG laser ablation, whereas nonsurgical treatment modalities are still under development. Focusing on AHs management in other districts, several treatment options have been documented including simple needle puncture, application of anticholinergic creams, trichloroacetic acid, and botulinum toxin, electrodessication, carbon dioxide laser vaporization, cryotherapy, and laser treatment [2, 4].

## 4. Conclusion

In case of a well-circumscribed, movable, exophytic submucosal mass with a yellowish appearance and no clinical signs of erythema or ulcerations, AH should be included in the differential diagnosis. Clinically, it appeared as a slow-growing asymptomatic lesion that could have followed a mucosal trauma. The histopathologic analysis is mandatory to obtain a final diagnosis due to its peculiar aspects. The surgical excision of the mass resulted in satisfying healing of the oral mucosa with no recurrence.
